# Magnetic resonance imaging enhancement of spinal nerve roots in a boy with X-linked adrenoleukodystrophy before diagnosis of chronic inflammatory demyelinating polyneuropathy

**DOI:** 10.1016/j.radcr.2023.10.065

**Published:** 2023-11-18

**Authors:** Derryl Miller, Laurence Walsh, Lisa Smith, Nucharin Supakul, Chang Ho, Toshihiro Onishi

**Affiliations:** a Department of Clinical Neurology, Indiana University School of Medicine, Indianapolis, IN, USA; b Department of Clinical Neurology, Genetics, and Pediatrics, Indiana University School of Medicine, Indianapolis, IN, USA; c Department of Clinical Radiology and Imaging Sciences, Indiana University School of Medicine, Indianapolis, IN, USA; d Department of Pediatrics Hematology/Oncology, Indiana University School of Medicine, Indianapolis, IN, USA

**Keywords:** Adrenoleukodystrophy, Very long chain fatty acids, ATP binding cassette subfamily D member 1, Chronic inflammatory demyelinating polyneuropathy

## Abstract

We present a boy with X-linked adrenoleukodystrophy (X-ALD) who was found to have lumbar nerve root enhancement on a screening MRI of the spine. The MRI was performed for lower extremity predominant symptoms. Several weeks after this MRI, he developed leg pain and was averse to walking long distances. He was diagnosed with Chronic Inflammatory Demyelinating Polyneuropathy (CIDP) with electromyography, nerve conduction studies, and serial imaging. His case is consistent with CIDP in association with X-ALD based on improvement with intravenous immunoglobulin (IVIG) with continued contrast enhancement and lower extremity symptoms 8 weeks after his initial scans. Contrast enhancement of nerve roots has not been previously described in X-ALD. Nerve root enhancement has been seen in other leukodystrophies such as globoid cell leukodystrophy and metachromatic leukodystrophy. This case also demonstrates comorbid X-ALD with CIDP and highlights possible mechanisms from the literature for this association. We also review the broad differential of cauda equina nerve root enhancement.

## Case report

**Fig. 1:**
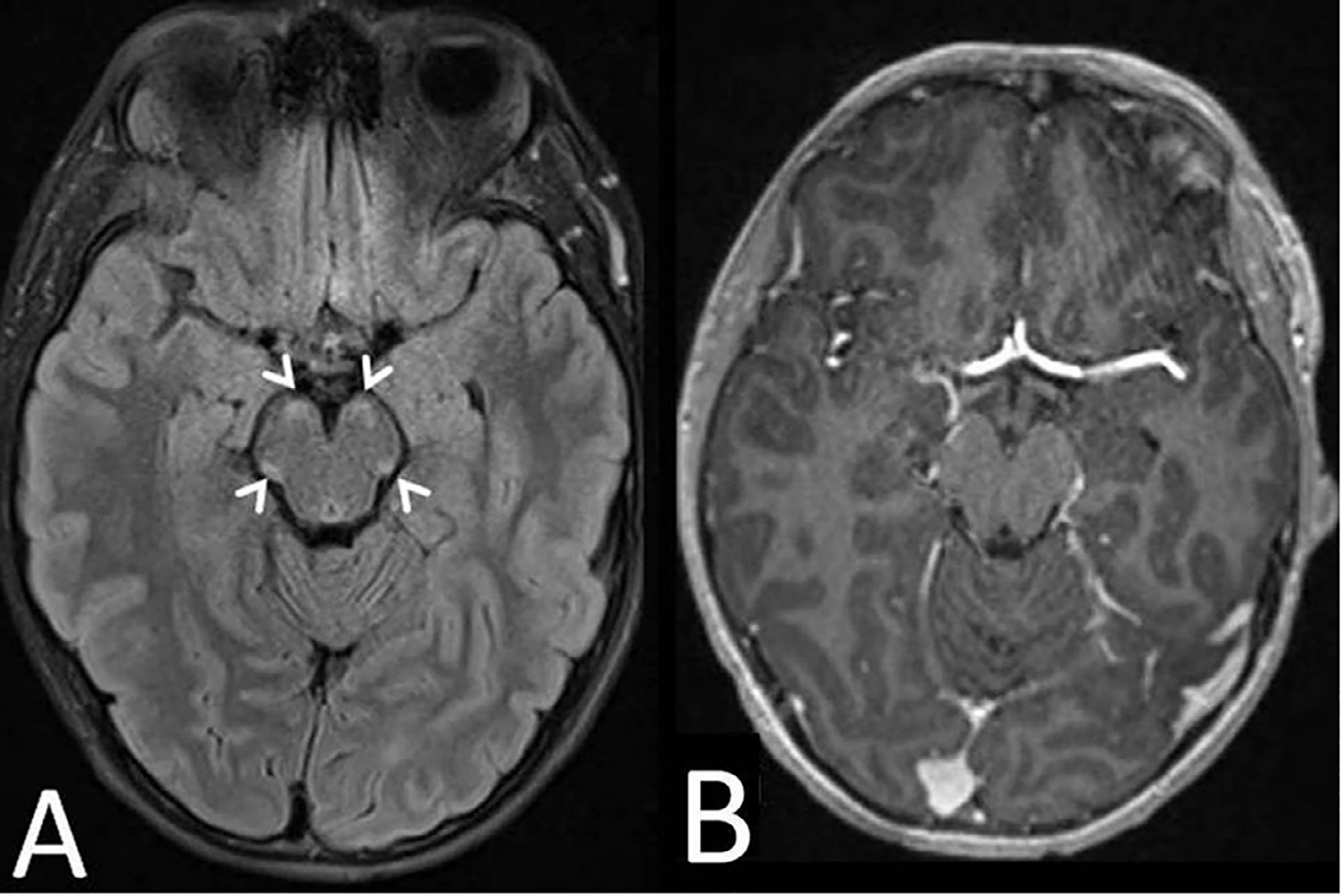
(A) Axial MRI brain without contrast on 9-20-19 with T2 fluid attenuated inversion recovery (FLAIR) with arrowheads showing hyperintense lesions of the cerebral peduncles. (B) Axial MRI brain with gadolinium contrast on 9-20-19 with T1 sequence at the level of the cerebral peduncles showing no contrast enhancement of these lesions.

**Fig. 2:**
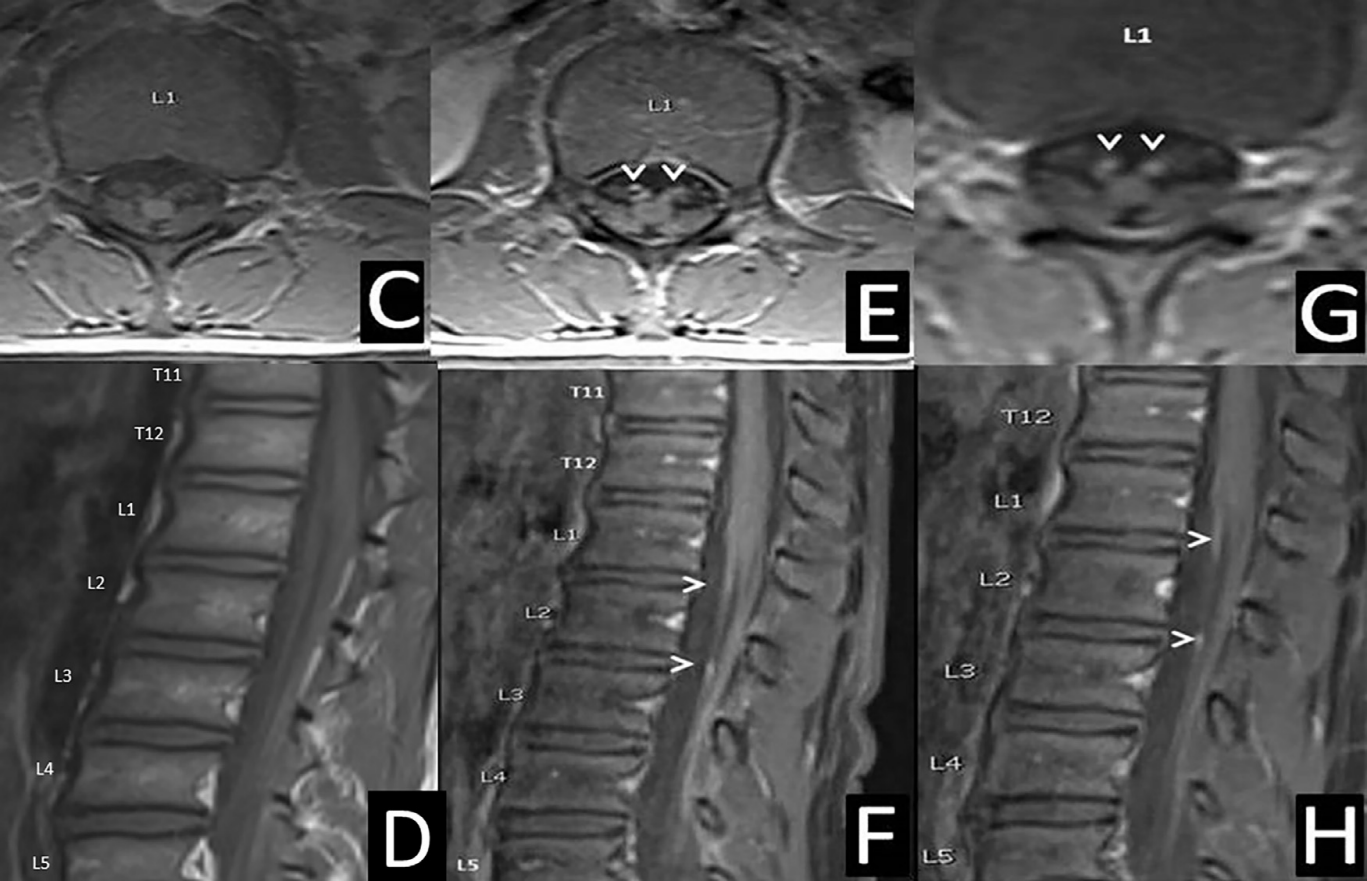
(C) Axial lumbar spine MRI on 3-12-20 with volumetric interpolated breath-hold examination (VIBE) without contrast. (D) Sagittal lumbar spine MRI on 3-12-20 with T1 turbo spin echo (TSE) showing termination and signal of the conus medullaris at L1. (E) Axial lumbar spine MRI on 3-12-20 with T1 VIBE postcontrast with arrowheads showing enhancing nerve roots. (F) Sagittal lumbar spine MRI on 3-12-20 with T1 TSE postcontrast with arrowheads showing enhancing nerve roots. (G) Follow-up axial lumbar spine MRI on 5-15-20 with T1 VIBE postgadolinium contrast with arrowheads showing enhancing nerve roots, though with decreased enhancement from the previous study. (H) Follow-up sagittal lumbar spine MRI on 5-15-20 with T1 TSE postgadolinium contrast with arrowheads showing enhancing nerve roots, though with decreased enhancement from the previous study.

A 15-year-old boy with adrenal insufficiency with an ATP Binding Cassette Subfamily D Member 1 (ABCD1) gene mutation presented to the neurology clinic following an abnormal screening MRI of the brain without and with gadolinium contrast. The MRI demonstrated fluid attenuated inversion recovery (FLAIR) hyperintense signal changes within the cerebral peduncles without contrast enhancement (Fig. 1). His sister and mother carry the mutation in ABCD1. His maternal grandmother was suspected to have adrenomyeloneuropathy with progressive disability and early death at age 56. Based on his MRI T2 FLAIR findings, his ALD MR Severity score was 2 [1]. He was referred for a stem cell transplant given mild clinical symptoms and new central nervous system involvement [1]. He is in a mainstream high school with an individualized education plan for extra testing time for attention deficit hyperactivity disorder (ADHD). Neuropsychiatric testing demonstrated full-scale IQ of 86 (low average) and a verbal comprehension index of 89 (low average). His neurologic exam showed evolving spasticity of the lower extremities, symmetric nonsustained ankle clonus, and mildly spastic gait with an ALD neurologic function scale (NFS) of 1 [2]. Due to his lower extremity predominant symptoms, MRI of the total spine without and with gadolinium contrast was performed. The MRI of the spine revealed cauda equina nerve root enhancement predominantly in the L1 and L3 levels with normal cord signal intensity, volume, and position of the conus medullaris (Fig. 2).

Weeks later at his Make-A-Wish trip, he developed pain in his bilateral gastrocnemius muscles which worsened until he avoided walking due to pain. He had hypoesthesia and paresthesia in his right foot and left hand without weakness. He did not have dysarthria, dysphagia, or respiratory distress. Reflexes were 2+ in the upper extremities, though severely diminished at the patellar and Achilles tendons bilaterally. Lumbar puncture was performed with total nucleated cell count of 3/mm^3^ (Ref 0-7), red blood cell count of 0/mm^3^ (Ref 0-5), glucose of 53 mg/dL (Ref 40-70), protein of 65 mg/dL (Ref 15-45), and negative culture and stain. Myelin basic protein from CSF returned was 1.63 ng/mL (Ref 0-5.5). Acute inflammatory demyelinating polyneuropathy (AIDP) was suspected, so intravenous immunoglobulin (IVIG) 2 g/kg was given divided over 5 days. Electromyography and nerve conduction studies (EMG/NCS) of the left upper and lower extremities indicated a length-dependent polyneuropathy with demyelinating features and multiple conduction blocks of both sensory and motor nerves (Table 1). He had improvement in his pain and walked after IVIG though had continued mild lower extremity pain and spastic gait.

**Table 1:** EMG/NCS on 4-16-20 showed sensory nerve conduction studies were nonreactive (NR) in the left sural nerve and left ulnar nerve antidromic sensory responses. Motor nerve conduction studies indicated nonreactive (NR) left peroneal nerve response as well as abnormal left median nerve response with prolonged distal latency at the wrist of 4.7 ms (normal < 4.4 ms) and slowed conduction velocity from elbow to wrist of 25.6 m/s (normal > 49 m/s). The amplitude of the median motor response was normal. Limited electromyography was performed due to patient tolerance of testing of the left anterior tibialis muscle revealing normal motor unit action potentials and rest activity.

Motor nerve conduction studies								
Nerve and site	Amp (mV)	Normal	CV	Normal	DL (ms)	Normal		
Peroneal (Fibular).Left								
Ankle	NR				NR	<6.5		
Knee	NR	>2.00		>44	NR			
Median. Left								
Wrist	9.1 mV				4.7 ms	<4.4		
Elbow	8.9 mV	>4.00	25.6 m/s	>49.0	12.5 ms			
Sensory nerve conduction studies								
Nerve and site	Amp (µV)			Normal	CV	Normal	Peak L (ms)	Normal
Sural. Left								
Calf	NR			>6			NR	<4.4
Ulnar antidromic. Left								
Wrist	NR			>10			NR	<3.0
Limited EMG examination								
Rest activity					Voluntary activity			
Muscle	INS	FIBS	Positive waves	FASC	DUR	AMP	POLY	MUAPs
Anterior tibialis. Left	NL	0	0	0	NL	NL	0	NL

Repeat imaging of the spine was performed 8 weeks later due to continued symptoms. The follow-up MRI revealed similar cauda equina nerve root enhancement consistent with chronic inflammatory demyelinating polyneuropathy (CIDP). He required serial doses of IVIG (Fig. 2). Following IVIG infusions, the patient had repeat MRI imaging of the brain, and his lesions were stable and again without contrast enhancement. Repeat EMG/NCS about 1 year later confirmed the presence of a moderate length-dependent demyelinating polyneuropathy which was improved from the previous study with temporal dispersion (Table 2). Whole exome sequencing revealed the known X-linked hemizygous mutation p.Gln590Ter (CAG>TAG): c. 1768 C>T in exon 7 in the ABCD1 gene, though no other mutations. This mutation is shared with the mother and is a pathogenic nonsense mutation with loss of function of the ABCD1 protein. The stem cell transplant was held for arrested cerebral X-ALD based on serial imaging and supported by his stable NFS [3]. He remains independently ambulatory. He requires continued clinical and radiographic screening for cerebral X-ALD [3].

**Table 2:** Repeat EMG/NCS on 3-8-21 showed sensory nerve conduction studies with nonreactive (NR) left sural nerve with normal left radial nerve response. Motor nerve conduction studies indicated left peroneal motor recorded at the extensor digitorum brevis was also NR, but present and low in amplitude at the anterior tibialis. There was decreased amplitude, prolonged latency, and decreased conduction velocity with temporal dispersion of the left tibial nerve. There was decreased conduction velocity and temporal dispersion of the left median nerve, though overall improved from the previous study. Blink reflexes were within normal limits. Limited needle EMG again demonstrated no abnormal rest activity and normal voluntary motor unit action potentials at the Anterior Tibialis and Vastus Medialis muscles.

Motor nerve conduction studies								
Nerve and site	Amp (mV)		Normal	CV	Normal	DL (ms)	Normal	
Peroneal (Fibular) Left								
Ankle	NR					NR	<6.5	
Peroneal (Fibular) Left – Tibialis anterior								
Knee	4.2 mV		> 5.1			6.41 ms	< 6.8	
Tibial Left – AH								
Ankle	1.8 mV		> 4.0	21.3 m/s	>41.0	8.02 ms	<5.8	
Knee	1.7 mV		> 4.0			26.3 ms		
Median left								
Wrist	10.3 mV			31.3 m/s	> 49.0	4.38 ms	< 4.4	
Elbow	10.0 mV		> 4.00			11.6 ms		
Sensory nerve conduction studies								
Nerve and Site	Amp (µV)	Normal	CV	Normal	Peak L (msec)	Normal		
Sural. Left								
Calf	NR	> 6			NR	<4.4		
Radial. Left								
Forearm	10.2 µV	>10			2.7 ms	<2.8		
Blink reflex								
Muscle	Stim side	R1 – Ipsi	NL	R2 – Ipsi	NL	R2 - Contra	NL	
Orbicularis Oculi								
	Left	11.2	U 13	37.4	U 40	37.8	U 44	
	Right	12.0	U 13	36.0	U 40	36.0	U 44	
Limited EMG examination								
Rest activity						Voluntary activity		
Muscle	INS	FIBS	Positive waves	FASC	DUR	AMP	POLY	MUAPs
Anterior Tibialis. Left	NL	0	0	0	NL	NL	0	NL
Vastus Medialis. Left	NL	0	0	0	NL	NL	0	NL

## Discussion

X-ALD is the most common peroxisomal leukodystrophy in childhood caused by an ABCD1 gene mutation on Xq28. Loss of function of ABCD1 results in failed transport of very long chain fatty acids (VLCFA) to the peroxisomes for beta oxidation [4,5]. Clinical manifestations of X-ALD include variable onset in childhood, adolescence, or adulthood with behavior changes, cognitive decline, adrenal insufficiency, weakness, spasticity, and bowel and bladder dysfunction [4,5]. On imaging and electrophysiologic studies, X-ALD presents variably with cerebral demyelination, projection fiber demyelination, myelopathy with atrophy of the spinal cord, and predominantly demyelinating peripheral neuropathy subtypes [4], [5], [6]. Active CNS disease is determined with clinical exam and confirmed with imaging studies with gadolinium contrast enhancement [1,3]. Contrast enhancement suggests inflammation with contrast extravasation due to disruption of the blood-brain barrier [1,3]. Cerebral demyelination in X-ALD commonly begins in the splenium of the corpus callosum with posterior predominance and relative sparing of the U-fibers of the cortex [1]. Rare variants of X-ALD have been described with anterior predominance, focal disease of the corticospinal tracts, and spinocerebellar predominant involvement [7,8]. Our patient did not have demyelinating lesions cranially to the corticospinal tracts of the midbrain in imaging studies, though he also had cauda equina nerve root enhancement on his MRI of the spine. Contrast enhancement of spinal nerves has not been reported in X-ALD. Cauda equina nerve root enhancement has previously been reported with metachromatic leukodystrophy and globoid cell leukodystrophy [9]. There is a report of AIDP in a patient with X-ALD, though this association is not common [10]. The patient's cerebrospinal fluid showed albuminocytological dissociation with normal cell count and elevated protein which is seen in AIDP, though albuminocytological dissociation is also seen in patients with X-ALD without AIDP [11,12]. The presence of albuminocytological disassociation does not predict a severe X-ALD course, and our patient reinforces this observation as his X-ALD is arrested [11]. Myelin basic protein in CSF has been shown to correlate with disease severity, and the patient's low normal result indicates a mild course [11]. Electrophysiologic abnormalities including conduction block in somatosensory evoked potentials and prolonged central motor conduction times in motor evoked potentials have been seen in female carriers of ABCD1 [13]. These patients were not evaluated with spine imaging to assess for nerve root contrast enhancement [13]. If cauda equina nerve root enhancement is seen, a broad differential including infectious, genetic, inflammatory, neoplastic, autoimmune, and demyelinating disorders must be considered. EMG/NCS can be helpful for narrowing this broad differential. EMG/NCS in adults with adrenomyeloneuropathy demonstrate predominance of demyelinating features affecting legs more than arms and motor nerves more than sensory nerves without temporal dispersion [14]. EMG/NCS in CIDP shows severe demyelinating features with temporal dispersion [15]. Our case is clinically and radiographically inconsistent with adrenomyeloneuropathy due to the absence of spinal cord atrophy, his young age of onset, and retained continence [3,6]. He could still develop adrenomyeloneuropathy in the future [3,6]. The duration of cauda equina nerve root enhancement in AIDP after the onset of symptoms has previously been reported to be up to 28 days in prospective cohort studies [16]. Our case had nerve root enhancement preceding the onset of his symptoms of AIDP and prolonged contrast enhancement beyond 28 days, suggesting that nerve root enhancement is seen with X-ALD in addition to other leukodystrophies. His persistent contrast enhancement after 8 weeks and improvement with IVIG indicates that he developed comorbid CIDP. X-ALD associated with CIDP was previously hypothesized to occur due to the association of autoimmune diseases, antiganglioside antibodies binding of VLCFA, and accumulation of VLCFA causing myelin instability [10]. Further study will be needed to formalize the relationship between the association of X-ALD and CIDP [10]. While IVIG is therapeutic for reducing the severity of the symptoms at the nadir of AIDP and for relapse of symptoms in CIDP, IVIG has not been shown to be effective in treating or arresting X-ALD [4].

## Conclusion

X-ALD is occasionally diagnosed comorbidly with AIDP or CIDP with more research being required to determine the exact cause of this association. Other leukodystrophies, AIDP, and CIDP have demonstrated cauda equina nerve root enhancement with gadolinium contrast on MRI of the spine. Our case suggests contrast enhancement is seen with X-ALD due to contrast enhancement being present prior to the onset of our patient's symptoms of CIDP. Clinical improvement with IVIG also suggests that the patient had CIDP with X-ALD. The neuroradiologist can consider a broad differential when contrast-enhancing cauda equina nerve roots are seen, including demyelinating conditions, neoplastic disorders, infectious etiologies, neurosarcoidosis, hereditary sensory and motor neuropathies, and leukodystrophies. A follow-up EMG/NCS can assist in narrowing this broad differential.

## Patient perspective

The patient noted improvement in his pain and numbness with IVIG infusion and again became independently ambulatory. He had several admissions for aseptic meningitis with headache and neck stiffness which were brief following intermittent infusion of IVIG 2 gm/kg for CIDP. Aseptic meningitis necessitated analgesia and intravenous fluids. He now is continuing surveillance for progression of X-ALD and wants to study to be a medical assistant. Over the last year, he has had improvement with physical therapy and occupational therapy and now ambulates and exercises well.

## Ethical statement

We confirm that we have read the Journal's position on issues involved in ethical publication and affirm that this report is consistent with those guidelines.

## Patient consent

Written consent was acquired from the family prior to drafting this manuscript.
